# Bridging pre-surgical endocrine therapy for breast cancer during the COVID-19 pandemic: outcomes from the B-MaP-C study

**DOI:** 10.1007/s10549-023-06893-4

**Published:** 2023-04-03

**Authors:** Rajiv V. Dave, Beatrix Elsberger, Vicky P. Taxiarchi, Ashu Gandhi, Cliona C. Kirwan, Baek Kim, Elizabeth M. Camacho, Charlotte E. Coles, Ellen Copson, Alona Courtney, Kieran Horgan, Patricia Fairbrother, Chris Holcombe, Jamie J. Kirkham, Daniel R. Leff, Stuart A. McIntosh, Rachel O’Connell, Ricardo Pardo, Shelley Potter, Tim Rattay, Nisha Sharma, Raghavan Vidya, Ramsey I. Cutress, B-MaP-C study collaborative, Abbas Imran, Abdalla Saad Abdalla Al-Zawi, Abeera Abbas, Ada Chrysafi, Adam Critchley, Adam Walsh, Ahmed Hamad, Ajay Sahu, Alex Knight, Alexandra Humphreys, Ali Salih, Alice Chambers, Alice Moody, Alsarah Diab, Amanda Taylor, Amanda Thorne, Amani Asour, Amit Agrawal, Amit Goyal, Amtul Carmichael, Amtul Sami, Andreas Larentzakis, Andrew Pieri, Angeline Tanhueco, Anita Hargreaves, Anita Sharma, Anjana Satpathy, Anna Heeney, Anna R. Hurley, Anne Tansley, Antonio Piñero-Madrona, Anu Sandhya, Anu Shrotri, Anup Sharma, Anushka Chaudhry, Anzors Gvaramadze, Aonghus Ansari, Arjun Kattakayam, Arnold D. K. Hill, Asha Adwani, Ashok Chouhan, Ashutosh Kothari, Ashvina Segaran, Atanu Ray, Bahar Mirshekar-Syahkal, Bahaty Riogi, Bashar Zeidan, Beatrix Elsberger, Bernadette Pereira, Brian Hogan, Brian Isgar, Carl Podesta, Carol-Ann Courtney, Caroline Mortimer, Caroline Pogson, Catherine Krzyzanowska, Cátia Felício, Channegowda Navin, Charles Zammit, Charlotte Ives, Charlotte Kallaway, Cheryl Lobo, Chloe Williams, Christiana Laban, Christopher W. J. Cartlidge, Christopher Wilson, Ciara McGoldrick, Ciaran Hollywood, Claire Louise Rutherford, Claudia Harding-Mackean, Claudiu Simonca, Colm Power, D. H. B. Ubayawansa, Dalia Elfadl, David Dodwell, David Mark Sibbering, David Rew, Deepika Akolekar, Demetrios Hadjiminas, Dennis Remoundos, Dheer Singh Rana, Diana Gonçalves, Dibendu Betal, Dibyesh Banerjee, Dinesh Thekkinkattil, Disha Mehta, Donna Egbeare, Dorin Dumitru, Douglas Ferguson, Duraisamy Ravichandran, E. Rahman, Edel Quinn, Edward R. C. St John, Eiman Khalifa, El-Rasheed Abdalla, Elaine Borg, Elaine Hyett, Eleanore J. Massey, Eleni Ntakomyti, Eleri Davies, Eliana Kalakouti, Elizabeth Clayton, Elizabeth Smyth, Ellora Barua, Emanuele Garreffa, Emma de Sousa, Emma MacInnes, Ennio Agabiti, Erum Najeeb, Evangelos Mallidis, Fabio Rapisarda, Farrokh Pakzad, Fathi Salem, Fayyaz Mazari, Firas Eddin Bachir Alkistawi, Frances Kenny, Frank Trollman, Gael MacLean, Gareth W. Irwin, George Boundouki, Georgette Oni, Georgios Exarchos, Georgios Karagiannidis, Gerald Gui, Geraldine Mitchell, Gerard Byrne, Gillian Clayton, Giulio Cuffolo, Giuseppina Mondani, Gordon Urquhart, Hannah Knowles, Haresh Devalia, Hazem Khout, Helen Dent, Helen M. Sweetland, Helen Mathers, Henrique Mora, Henry Cain, Henry Douglas Robb, Hiba Fatayer, Hisham Hamed, Hudhaifah Shaker, Hussein Tuffaha, Hyunjin Shin, Iain Brown, Ian Daltrey, Ian Whitehead, Ian Young, Iman Azmy, Imran Abbas, Inga Peerlink, Irene Athanasiou, Irene Osorio-Silla, Isabella Dash, James Bentley, James Cook, James Harvey, Jamie McIntosh, Jamie Vatish, Jane Aitken, Jane Ooi, Jane Ralph, Jane Turner, Jarin Louis Noronha, Jaroslaw Krupa, Jasdeep Gahir, Jasper Gill, Jennifer E. Rusby, Jennifer Isherwood, Jennifer Long, Jennifer Peck, Jenny Banks, Jeremy Batt, Jibril Jibril, Jo Mondani, Joanna Franks, Joanna Seward, John Benson, John Murphy, Jonathan D. Horsnell, Jonathan Lund, Jonida Selmani, Jose I. Sanchez-Mendez, Joseph Maalo, Julia Henderson, Julia Massey, Julie Doughty, Julie Dunn, Kalliope Valassiadou, Kamal Pushdary, Karen Bosch, Karen James, Karina Cox, Karyn Shenton, Kat McEvoy, Katalin Zechmeister, Katarina Lakatosova, Kate E. Williams, Katharine Kirkpatrick, Katherine Fairhurst, Katherine Krupa, Kathryn E. Harborough, Katy Hogben, Kelly Lambert, Kelvin Chong, Khalid Amin, Kristjan Asgeirsson, Kwok-Leung Cheung, Laszlo Romics, Lee Martin, Lee-Min Lai, Leena Chagla, Lisa Whisker, Loraine Kalra, Lorna Cook, Louise Alder, Louise Merker, Lubna Noor, Lucie Jones, Lucy R. Khan, Lydia Prusty, Lyndsey Highton, M. Bilal Elahi, Maged Hussien, Mairead Savage, Mairi Fuller, Manoj Gowda, Maria Bramley, Maria Callaghan, Maria Vernet-Tomas, Maria Verroiotou, Marta Jimenez Gomez, Massimiliano Cariati, Masuma Sarker, Matthew Hague, Matthew Rowland, Michael Faheem, Michael J. Allen, Michelle Mullan, Mike Shere, Mina Girgis, Mina M. G. Youssef, Mini V. Sardar, Mohamed Elamass, Mohamed Elkorety, Mohamed Lafi, Mohamed Razick Sait, Mohammad Amir Sharif, Mohammed Absar, Mohsin Dani, Mona Sulieman, Monika Kaushik, Muhammad Salman, Musa Barkeji, Mysore Chandrashekar, Nabila Nasir, Nader Touqan, Nadine Betambeau, Nathan Coombs, Neil Johns, Neill Patani, Ngee-Ming Goh, Nicholas Gallegos, Nicholas Holford, Nick Abbott, Nicola Barnes, Nicola Laurence, Nicola Roche, Nikitas Dimopoulos, Nikolaos V. Michalopoulos, Norah Scally, Noyko Stanilov, Nur Amalina Che Bakri, Oladapo Fafemi, Olubunmi Odofin, Panagiotis Kokoropoulos, Pankaj Roy, Parto Forouhi, Paul Thiruchelvam, Pawel Trapszo, Penelope McManus, Peter A. Barry, Peter Liptay-Wagner, Peter Mallon, Petros Charalampoudis, Philip Drew, Philip Turton, Pilar Matey, Polly King, Polly Partlett, Primeera Wignarajah, Rachel Ainsworth, Rachel Elizabeth English, Rachel Foster, Rachel Soulsby, Rachel Tillett, Rachel Xue Ning Lee, Radhika Chadha, Ragheed Al-Mufti, Raj Achuthan, Raja Eid, Rajaram Burrah, Rajiv Vashisht, Rajive Nair, Ralia Bunza, Raman Vinayagam, Rami Tabbakh, Raouef Ahmed Bichoo, Rathi Rathinaezhil, Rebekah Law, Reem Salman, Reginald Salvador, Riccardo Bonomi, Richard Johnson, Richard Sutton, Rishikesh Parmeshwar, Ritchie Chalmers, Ritika Rampal, Rob Hardy, Robert Macmillan, Robert Thomas, Rogelio Andrés-Luna, Rosamond Jacklin, Rosie Simson, Russell Mullen, Ruth James, Ruvinder Athwal, Sa’ed Ramzi, Sabrina Bezzaa, Sadaf Jafferbhoy, Sam Jeffreys, Samantha A. Sloan, Samantha K. Williams, Samir Laali, Samy Shaheed, Sanjay Joshi, Sankaran Chandrasekharan, Sankaran Narayanan, Santosh Somasundaram, Sarah Barker, Sarah Horne, Sascha Dua, Sasi Govindarajulu, Saung Hnin Phyu, Sekhar Marla, Senthurun Mylvaganam, Shabbir Poonawala, Shamaela Waheed, Sharat Chopra, Sharon Wallace, Sheila Shokuhi, Sheila Stallard, Sherif Monib, Shireen Mckenzie, Simon Harries, Simon Hawkins, Simon Marsh, Simon Pain, Simon Pilgrm, Simon Smith, Simon Thomson, Siobhan Rooney, Sisse Olsen, Soni Soumian, Sonia Bathla, Stacy Wardle, Stephanie C. Jenkins, Stephen McCulley, Stuart Robertson, Sumit Goyal, Sumohan Chatterjee, Sunita Saha, Susan Williams-Jones, Syeda Nadia Shah Gilani, Tamara Kiernan, Tania S. de Silva, Tapan Sircar, Tasha Gandamihardja, Theodoros A. Sidiropoulos, Thomas Stroud, Tin Aung Sein, Toral Gathani, Tracey Irvine, Tuabin Rasheed, Urvashi Jain, Usama Suleiman, Uzma Andaleeb, Vallipuran Gopalan, Vasileios Sakellariou, Venla Kantola, Vinod Mathen, Wail Al Sarakbi, Walid Sasi, Wendy Sotheran, William H. Allum, Yasmin Wahedna, Yazan Masannat, Youhana Mikhael, Yousuf Sabah, Zaid Al-Ishaq, Zarghuna Taraki, Zenon Rayter, Abigail Tomlins, Alda Correia, Amir Sharif, André Magalhães, Anjana Sathpathy, Antonio Piñero Madrona, Asma Al-Allak, Aurea Manso de Lema, Bashar Zedian, Balendra Kumar, Brendan Smith, C. Navin, Caroline Richardson, Chandra Sekharan, Chloe Constantinou, Chris Wayte, Christina Summerhayes, Clare Fowler, Claire Murphy, Colin Rogers, Covadonga Marti Alvarez, Douglas Macmillan, Eamonn Coveney, Eleanor Gutteridge, Eleftheria Kleidi, Elisa York Pineda, Fernando Osório, Fiona Court, Francis Kenny, Gary Osborn, Georgina Yiasoumis, Gloria Petralia, Harleen Deol, Richard Hunt, John Robertson, José Luis Fougo, Lara Miralles Olivar, Laura Johnson, Mahwash Baber, Marcel Segura Badia, M. D. Zaker Ullah, D. Hassanally, Nicola Dunne, Susie Connolly, Mohsin El-Gammal, Brendan Skelly, Ibrahim Ahmed, P. W. Crane, Lucy Satherley, Tracey Simoes, Natarajan Vaithilingam, Nikolaos Arkadopoulos, Nikolaos Danias, Nuria Argudo, P. Macmanus, Pantelis Vassiliu, Pau Nicolau Batalla, Pilar Zamora Auñon, Rachel Tillett, Sarah B. Vestey, Sarah Tang, Sergio Salido, Shweta Aggarwal, Simon Pilgrim, Susy Costa, Zoe Winters

**Affiliations:** 1 The Nightingale Breast Cancer Centre, Wythenshawe Hospital, Manchester University NHS Foundation Trust, Manchester, M23 9LT UK; 2 Division of Cancer Sciences, School of Medical Sciences, Faculty of Biology, Medicine and Health, University of Manchester, Oglesby Cancer Research Building, Manchester Cancer Research Centre, Wilmslow Road, Manchester, M20 4BX UK; 3 Aberdeen Royal Infirmary/University of Aberdeen, Breast Unit, Foresterhill Road, Aberdeen, AB25 2ZN UK; 4 Division of Population Health, Health Services Research, and Primary Care, School of Health Sciences, University of Manchester, Manchester, M13 9PL UK; 5 Department of Breast Surgery, St. James’s University Hospital, Leeds, LS9 7TF UK; 6 Department of Oncology, University of Cambridge, Cambridge, UK; 7 Cancer Sciences Academic Unit, University of Southampton and University Hospital Southampton, Tremona Road, Southampton, SO16 6YD UK; 8 Department of Surgery and Cancer, Imperial College, London, UK; 9 Trustee, Independent Cancer Patients Voice, London, UK; 10 Linda McCartney Centre, Royal Liverpool and Broadgreen University Hospital, Prescot Street, Liverpool, L7 8XP UK; 11 Patrick G. Johnston Centre for Cancer Research, Queen’s University Belfast, 97 Lisburn Road, Belfast, BT9 7AE UK; 12 Department of Breast Surgery, The Royal Marsden NHS Foundation Trust, Downs Road, Sutton, Surrey, SM2 5PT UK; 13 Bolton NHS Foundation Trust, Minerva Rd, Farnworth, Bolton, BL4 0JR UK; 14 Bristol Centre for Surgical Research, Population Health Sciences, Bristol Medical School, Canynge Hall, Whatley Road, Bristol, BS8 2PS UK; 15 Bristol Breast Care Centre, North Bristol NHS Trust, Southmead Road, Bristol, BS10 5NB UK; 16 Leicester Cancer Research Centre, Clinical Sciences Building, University of Leicester, Leicester, LE2 2LX UK; 17 Breast Unit, Level 1 Chancellor Wing, St James’s Hospital, Leeds, LS9 7TF UK; 18 The Royal Wolverhampton NHS Trust, Wolverhampton Road, Wolverhampton, WV10 0QP UK

**Keywords:** COVID-19, Breast cancer, Neoadjuvant endocrine therapy, Bridging endocrine therapy

## Abstract

**Purpose:**

The B-MaP-C study investigated changes to breast cancer care that were necessitated by the COVID-19 pandemic. Here we present a follow-up analysis of those patients commenced on bridging endocrine therapy (BrET), whilst they were awaiting surgery due to reprioritisation of resources.

**Methods:**

This multicentre, multinational cohort study recruited 6045 patients from the UK, Spain and Portugal during the peak pandemic period (Feb–July 2020). Patients on BrET were followed up to investigate the duration of, and response to, BrET. This included changes in tumour size to reflect downstaging potential, and changes in cellular proliferation (Ki67), as a marker of prognosis.

**Results:**

1094 patients were prescribed BrET, over a median period of 53 days (IQR 32–81 days). The majority of patients (95.6%) had strong ER expression (Allred score 7–8/8). Very few patients required expedited surgery, due to lack of response (1.2%) or due to lack of tolerance/compliance (0.8%). There were small reductions in median tumour size after 3 months’ treatment duration; median of 4 mm [IQR − 20, 4]. In a small subset of patients (*n* = 47), a drop in cellular proliferation (Ki67) occurred in 26 patients (55%), from high (Ki67 ≥ 10%) to low (< 10%), with at least one month’s duration of BrET.

**Discussion:**

This study describes real-world usage of pre-operative endocrine therapy as necessitated by the pandemic. BrET was found to be tolerable and safe. The data support short-term (≤ 3 months) usage of pre-operative endocrine therapy. Longer-term use should be investigated in future trials.

**Supplementary Information:**

The online version contains supplementary material available at 10.1007/s10549-023-06893-4.

## Introduction

In order to navigate the risk posed by the COVID-19 pandemic, changes were introduced to breast cancer care due to rationalisation of resources and prioritisation of individual patient’s cancer risks versus COVID-19 risks [1–7]. This occurred primarily during the first wave of infections, when the true impact of COVID-19 was unknown, vaccines were not yet available, and measures were introduced to maintain patients’ safety against the virus. This had potential to impact cancer care survival and quality of life (QoL) during the COVID-19 pandemic, but also offered a unique opportunity to prospectively observe the impact of non-standard care.

GlOBOCAN 2020 data shows that breast cancer represents 12% of all cancers diagnosed in Europe [8], and with current treatments early breast cancer prognosis is usually excellent. The management of patients diagnosed with primary breast cancer during the pandemic aimed to maintain these high standards of care whilst healthcare resources were re-prioritised to deal with the virus. This was challenging, as hospitals were considered a high-infection risk environment, and the multi-modal treatment of breast cancer involves multiple hospital visits, including surgery, radiotherapy (RT), and systemic therapy. Multidisciplinary UK, European and American guidelines, were published early in the alert phase, which helped to inform management of breast cancer during the pandemic [1, 4–6, 9–11]. The majority of recommendations did not deviate substantially from pre-COVID breast cancer management international guidance [12], but highlighted less commonly utilised alternatives, emphasizing treatment pathways that aimed to reduce the risk of and exposure to SARS-CoV-2 infection. Where theatre capacity was compromised, breast cancer treatment guidelines included use of pre-operative, or ‘bridging’, endocrine therapy (ET) [3] and priority-driven surgery scheduling based on tumour biology [6, 7, 11].

The B-MaP-C study [13, 14] investigated the changes to routine or ‘standard’ breast cancer management pathways during the first wave of the pandemic in Europe, such as alterations to use of chemotherapy (neoadjuvant and adjuvant) [11] and streamlined use of adjuvant radiotherapy (RT) [15] including the use of hypofractionated, or, 5 fraction radiotherapy (5F RT). The most frequently utilised intervention to mitigate the expected delays during the pandemic was ‘bridging’ endocrine therapy (BrET) as initial treatment for those patients with oestrogen-receptor (ER) positive cancers whilst awaiting available operating theatre capacity. The first published results of the B-MaP-C study showed that in the UK subset, of the 2216 patients who had modified MDT decisions in the pre-operative setting, 951 patients received BrET [13]. In this follow-up study, we investigated in more detail the practice and outcomes of BrET use during this period in a real-world setting across three European countries, including its effect on imaging utilisation, disease downstaging, change in surgical plan, and correlation of BrET duration with downstaging (lesion size) and tumour proliferation (Ki67).

## Material and methods

The B-MaP-C study was a multicentre multinational cohort study which examined treatment recommendations during the first peak of the COVID-19 pandemic (defined as 1st February 2020 to 31st July 2020). Consecutive patients with a diagnosis of early breast cancer (invasive and DCIS) undergoing MDT-guided management were eligible for inclusion, and were identified prospectively by the local participating clinical teams in the UK, Greece, Spain and Portugal. Collection of patient demographic data, cancer-specific data and multi-disciplinary treatment recommendations in the pre-operative, operative and post-operative setting has previously been described [16, 17]. Management decisions made in the multi-disciplinary meeting (MDM) were considered as ‘standard’ if this would have been recommended pre-COVID for that particular unit, or ‘COVID-altered’ if this management recommendation was altered from that unit’s usual practice because of the COVID-19 pandemic. A ‘COVID-altered’ management decision included placing patients on BrET at the time of diagnosis; this includes patients with large hormone-receptor positive cancers having pre-operative ET, if this falls out with the unit’s standard practice. If patients had ‘standard’ treatment, no further clinicopathological data were collected other than tumour stage. Demographic, clinical (including radiological) and pathological data were captured at the time of the MDM decision to commence BrET. Patients were followed until treatment was completed or the close of the study in December 2020. Data collected included;

(i) date of surgery (and hence length of BrET) and whether this was; (a) a planned date on commencement of BrET or when theatre capacity became available; (b) surgery expedited due to lack response or intolerance of BrET; or (c) continuation on ET as the primary treatment option.
(ii) whether a change in surgical plan was instigated in patients with planned mastectomy as reported by the collaborator, and whether BCS was actually performed, or the patient chose to have a mastectomy
(iii) whether any imaging was performed during BrET to assess response, and the reported invasive size
(iv) the final pathological invasive size on the surgical excision, and whether a repeat Ki67 was performed. Ki67 change was categorised in accordance with published data as low–low (Ki67 before and after treatment < 10%); high–low (Ki67 before ≥ 10%, Ki67 after < 10%); high–high (Ki67 before and after ≥ 10%); and low–high (Ki67 before < 10%, Ki67 after ≥ 10%) [16].

We assessed response to BrET in those with invasive disease (excluding those with DCIS only) using the baseline ultrasound (USS) assessment of size, against (i) USS performed whilst on BrET and (ii) final post-surgical pathological size. The rationale behind this assumption is, that a study of the practice of BCS using image-guided localisation [17] prior to the pandemic where no delay in surgery is expected, has shown that the pathological size is marginally larger than the USS size (a median increase of 2 mm [IQR − 1, 7], *N* = 1521 patients).

Data were collected and managed using REDCap™ electronic data capture tools hosted at The University of Manchester [18], in accordance with Caldicott II principles. Prior to commencement of data collection, participating units were required to register the study locally with their hospital audit department and obtain local governance approvals. Ethics approval was not required according to the NHS Health Research Authority online decision tool (www.hra-decisiontools.org.uk/research/).

### Data analysis

The study was reported in accordance to the STROBE guidelines for observational studies [19]. A pre-specified statistical analysis plan was approved by the study steering group. Descriptive analysis examined characteristics of patients in whom standard management was followed and in those who had BrET. Continuous variables are presented by means (standard deviation, SD) or medians (interquartile range, IQR), categorical variables are presented by frequency (percentage). Calculations for each categorical variable were performed following exclusion of missing values for this variable only. Non-parametric Mann–Whitney tests were performed to test for differences of medians between groups separately for each continuous and ordinal variable and Chi-squared tests for associations between nominal variables. Analyses were computed using Stata MP (version 16).

## Results

**Fig. 1:**
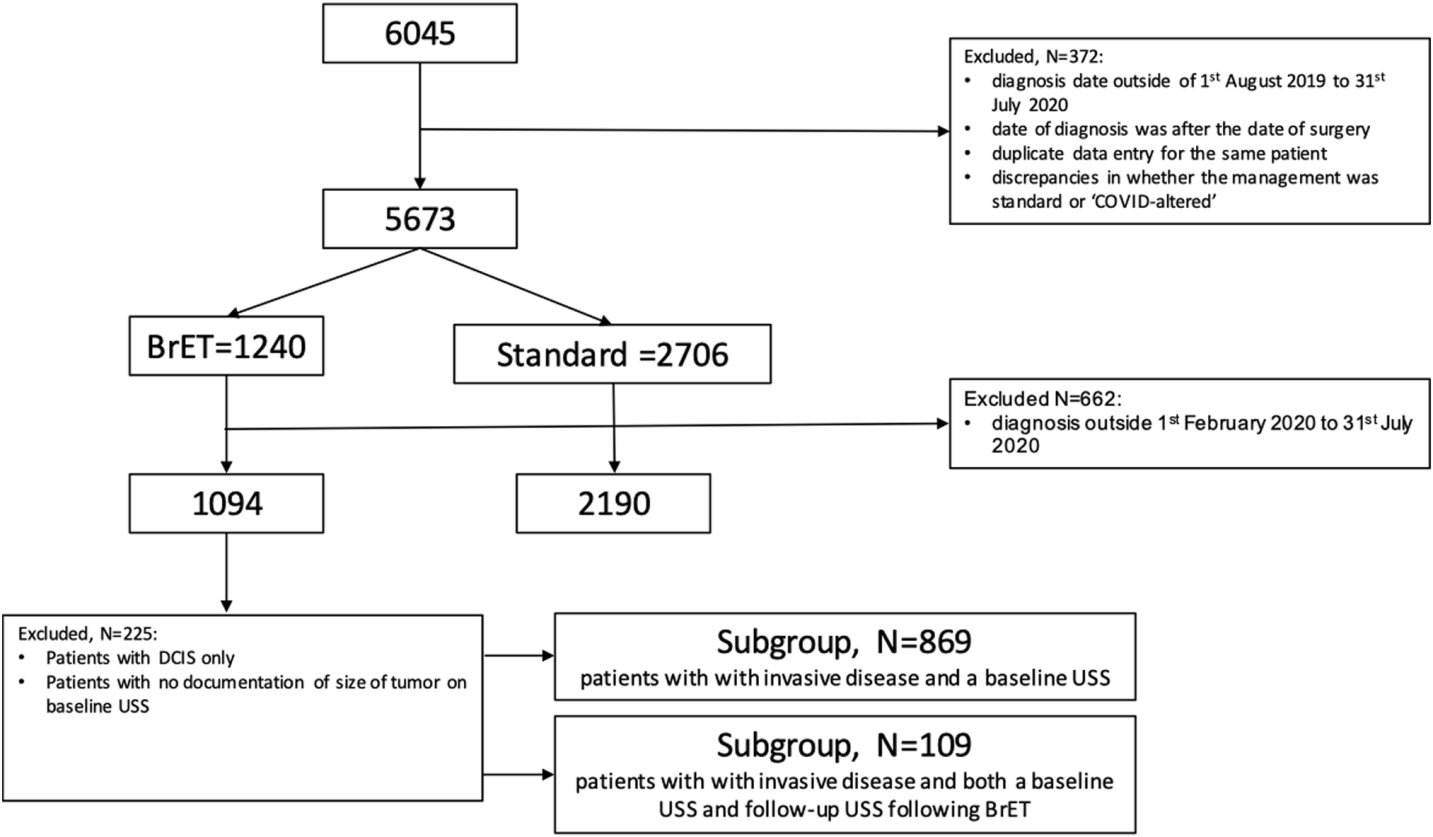
Consort diagram summarising the inclusion of patients in this study

There were 6045 patients included in the B-MaP-C study (Fig. 1), including patients from UK (*n* = 5378), Spain (*n* = 269) and Portugal (*n* = 26). Patients’ records were excluded if the date of diagnosis was outside of the study period, if the stated date of diagnosis was after the date of surgery, there was duplicate data entry for the same patient, and where there were discrepancies in whether the management was standard or ‘COVID-altered’. There were 5673 records available for final analysis, of which 1094 patients were prescribed BrET and 2190 patients had ‘standard’ management.

### Trends in management decisions

**Fig. 2:**
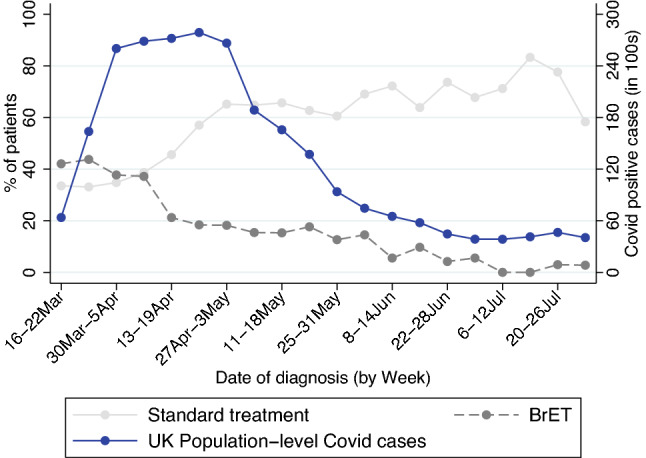
Use of BrET over time, compared to COVID-19 diagnosis, March–July 2022. Figure shows the changing trend in use of BrET over time (date of breast cancer diagnosis) showing UK data only. Data on COVID-19 diagnoses over period 16th March to 31st July 2022 from: https://coronavirus.data.gov.uk/details/cases

We examined the trend in ‘standard’ vs ‘BrET management decisions over time, compared to the number of positive COVID cases in the UK (data from https://coronavirus.data.gov.uk/details/cases). Figure 2 displays this data for the UK only, due to availability of raw data on COVID cases. At the start of the study, in March 2020, 40% of patients were placed on BrET, whereas by July 2020, after the end of the first COVID-19 wave in the UK, this reduced to 1%.

### Patients on ‘Bridging’ ET

Of the 1094 patients in whom BrET was prescribed; 179 (17%) patients were pre- or perimenopausal, but the majority were post-menopausal (871; 83%). Menopausal status was not available for 44 patients. Patients receiving BrET were older, and of lower stage compared to those receiving standard treatment (T1; 51% vs 36% and N0; 84% vs 70%) (Table 1). The majority of patients having BrET (95.6%) had strong ER positivity (ER 7–8/8 *Allred score; Table 1).

**Table 1:** Clinicopathological characteristics of patients included in the study

		Standard management	BrET	*p*-value	Total
		*N* = 2190	*N* = 1094		3284
Age (median-IQR)		58 (49–71)	66 (56–74)	< 0.001	61 (51–72)
	Missing	0	0		
T (*n* = 3247)	Tis	161 (7%)	95 (9%)	< 0.001	256
	T1	774 (36%)	557 (51%)		1311
	T2	931 (48%)	372 (34%)		1303
	T3	221 (11%)	58 (4%)		279
	T4	71 (4%)	7 (1%)		78
	Missing	32	5		
*N* (*n* = 3237)	N0/N1mi	1503 (70%)	911 (84%)	< 0.001	2414
	N1	457 (21%)	144 (13%)		601
	N2	114 (19%)	17 (2%)		131
	N3	79 (13%)	12 (1%)		91
	Missing	37	10		
M (*n* = 3274)	M0/MX	2118 (98%)	1080 (100%)	0.001	3198
	M1	40 (2%)	5 (0%)		45
	Missing	32	9		
ER (Allred score)	Negative (0–3)	n/a^a^	5 (1%)		
	Borderline (4–6)	n/a^a^	33 (4%)		
	Strong (7–8)	n/a^a^	838 (95%)		
WHO performance status (*n* = 3274)	0	1641 (75%)	767 (70%)	0.016	2408
	1	325 (15%)	225 (21%)		550
	2	131 (6%)	81 (7%)		212
	3	72 (3%)	18 (2%)		90
	4	14 (1%)	0 (0%)		14
	Missing	7	3		
Presentation (*n* = 3223)	Symptomatic	464 (22%)	464 (43%)	< 0.001	928
	Screen-detected	1675 (78%)	620 (57%)		2295
	Missing	51	10		

T, N are pathological TNM, except where patients were having ET/NACT, in which case TNM is taken from imaging, using T stage based on the largest reported size from all imaging modalities

*M1* patients who were diagnosed with metastatic disease after surgery, *T* tumour stage, *N* nodal stage, *M* metastases, *WHO* World Health Organisation

^a^Data not available

Of the 179 women classified as pre/peri menopausal and receiving BrET, 132 were prescribed Tamoxifen, 9 were prescribed ovarian suppression alone and 38 prescribed an aromatase inhibitor (AI). Of those 38 patients prescribed an AI, 12 were also prescribed ovarian suppression. Of the post-menopausal women, 96% (829/867) were prescribed an AI, with the remainder prescribed Tamoxifen (supplementary Table S1).

### Planning for surgery

**Fig. 3:**
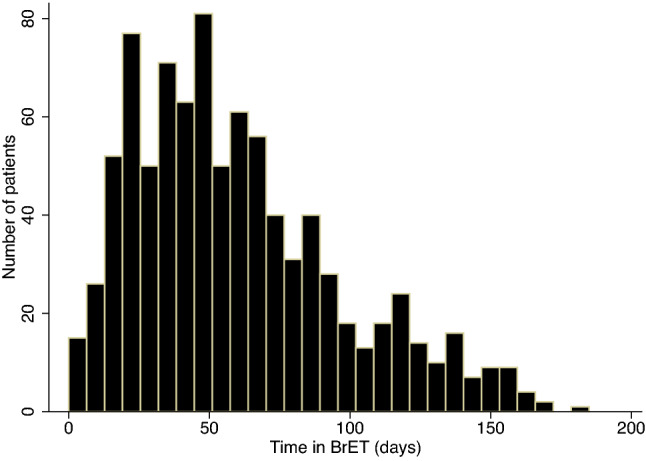
Length of time on BrET. Figure shows the length of time on BrET, determined as the time between prescription of BrET and date of surgery, in days

Of the 713 patients planned for breast-conserving surgery, 632 (88.6%), had a clip- placed in the tumour (81 no clip sited, 11 were unknown). The median length of BrET in those that had surgery (990 patients, data missing in *n* = 104) was 53 days (IQR [32, 81]) (Fig. 3). Of these, 28.5% (282/990) had their procedure expedited due to availability of theatre capacity earlier than expected. There were 1.2% (12/990) patients where surgery was expedited due to lack of response and 0.8% (8/990) where surgery had to be expedited due to lack of tolerance/compliance with BrET. There were 8.2% (81/990) in whom ET was continued as the primary treatment approach, mostly due to patients declining surgery (31/79, missing data in *n* = 2, Table 2).

**Table 2:** Reasons for patients continuing on ET which became their primary treatment modality

	*N* (%)
Patient not fit for surgery, but had response on ET	31 (39.2%)
Patient fit for surgery, but has chosen to decline surgery	31 (39.2%)
Other	17 (21.5%)
Total	79

### Imaging response to ‘Bridging’ ET

Additional post diagnostic follow-up imaging prior to surgery was undertaken in 151 (13.8%) patients on BrET. Of these 135 (89%) had ultrasound, 23 (15%) had mammogram, and 14 (9%) had MRI. Of those 135 patients who had a follow-up USS, there were 109 patients (with invasive disease only, excluding those with DCIS only) in whom data were available to assess response to BrET, using a baseline USS assessment of size against USS assessment whilst on BrET. Reductions in median USS size were small and were greater in post-menopausal than pre/perimenopausal women (− 3 mm [IQR − 7.5, 0], respectively vs − 1 mm [IQR − 3.7, 0]), and were only evident following more than 3 months BrET (median − 3 mm [IQR − 8, 0.5]) (Table 3).

**Table 3:** Objective response to BrET, Response to BrET as determined by the size on pathology compared to size on baseline USS (at diagnosis), and follow-up USS (post-BrET, pre-operative), Median [IQR]

	Difference in USS size (mm) between pre-operative USS whilst on BrET, and baseline diagnostic USS^a^ Median [IQR]	Difference in size (mm) between final; pathological size and baseline diagnostic USS^b^ Median [IQR]
Total	*N* = 109	*N* = 869
Menopausal status		
Pre or peri	− 1 [− 3.7, 0] (*n* = 15)	2 [− 2, 9] (*n* = 141)
Post	− 3 [− 7.5, 0] (*n* = 89)	0.5 [− 5, 7] (*n* = 695)
missing	5	33
Length of time on BET		
Up to 1 month	0 [− 1, 0] (*N* = 6)	2 [− 1, 8] (*N* = 179)
Up to 2 months	− 2 [− 4, 0] (*N* = 19)	2 [− 1, 8] (*N* = 256)
Up to 3 months	− 3.5 [− 12.5, 2.5] (*N* = 12)	2 [− 1, 9] (*N* = 163)
More than 3 months	− 3 [− 8, 0.5] (*N* = 72)	− 4 [− 20, 4] (*N* = 271)

^a^Patient-level data. A limited number of patients had a pre-operative USS following BrET and prior to surgery

^b^Patient-level data. (Size on Pathology, mm) − (size on USS, mm) (patient level data), where a negative difference reflects a reduction in size

### Change in surgical plan

Of the 1094 patients commenced on BrET, 713 (65%) had a stated baseline surgical plan for BCS (± mastectomy as per patient choice), and 275 (25%) for mastectomy only. The baseline surgical plan was not stated in 106 patients (10%). To assess whether there was a change in surgical plan whilst on BrET, collaborators were asked to document the type of breast surgery performed post-BrET. Of 275 patients who had a baseline plan for a mastectomy, 64% (178/275) proceeded with the planned mastectomy, whilst 35.2% (97/275) after BrET had their surgical options changed from the initial plan of mastectomy to, also include breast conservation. Of these, 13.4% (13/97) patients who elected to proceed with the initially planned mastectomy although it was stated that they were considered suitable for breast conservation post-BrET. The clinicopathological data in these groups are summarised in Tables 4.

**Table 4:** Clinicopathological variables comparing patients who had an initial surgical plan for a mastectomy

	Proceeded with planned mastectomy	Change in stated surgical plan, from mastectomy to accommodate breast conservation	Stated that achieved the potential for breast conservation, but treated by mastectomy
	*N* = 178 (%)	*N* = 84 (%)	*N* = 13 (%)
Length of ET Median [IQR]	50 [30, 79]	62 [36.5, 94.5]	92 [48, 132]
Pre-peri menopause, *n*(%)	38 (22.3)	13 (15.8)	0
Post menopause, *n*(%)	132 (77.7)	69 (84.1)	13 (100)
Size (mm) of invasive disease on diagnostic Mammography^a^ mean (sd)	27.7 (19.1)	21.4 (13.7)	20.9 (15.4)
Size (mm) of invasive disease on diagnostic Ultrasound^a^, mean (sd)	24.3 (16.7)	16.7 (10.7)	19.5 (11.2)
Size (mm) of invasive disease on pathological excision^a^	33.0 (23.7)	18.4 (10.3)	21.4 (10.8)
Grade = 1, *n*(%)	29 (16.4)	21 (25.3)	3 (23.1)
Grade = 2, *n*(%)	111 (62.7)	46 (55.4)	8 (61.5)
Grade = 3, *n*(%)	37 (20.9)	16 (19.3)	2 (15.4)
ER pos, *n*(%)	176 (98.9)	83 (98.8)	13 (100)
PR pos, *n*(%)	124 (84.9)	60 (84.5)	9 (81.8)
HER2 pos, *n*(%)	9 (5.5)	7 (9.6)	1 (8.3)
Ki67, median [IQR]	17 [9.5, 33.3]	12.5 [6.5, 18]	24.5 [19, 53]
Number of axillary node macrometastases, median [IQR]	0 [0, 1]	0 [0, 0]	0.5 [0, 1]

^a^Size is for invasive disease only, and does not take into account extent of DCIS

### Pre-operative imaging and pathology assessment of response to ‘Bridging’ ET

There were 869 patients (with invasive disease only, excluding those with DCIS only) in whom data were available to assess response to BrET, using a final post-surgical pathological size against baseline USS assessment of size. A reduction in invasive disease size on final pathology compared to pre-operative USS was only evident after 3 months of BrET (median − 4 mm [IQR − 20, 4]) (Table 3).

### Impact on tumour proliferation (Ki67) of BrET

**Fig. 4:**
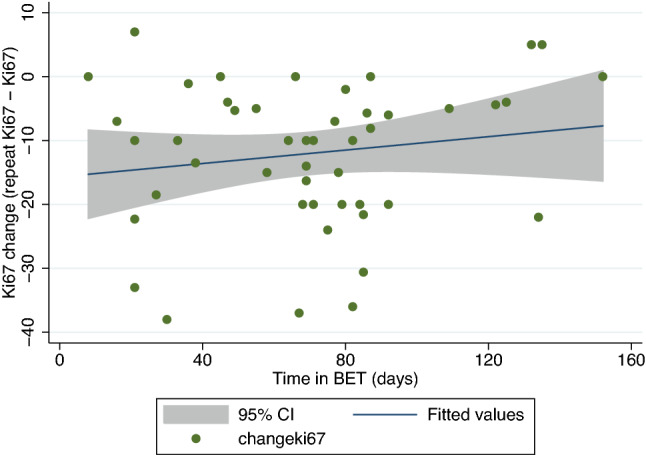
Absolute change in Ki67 against time on BrET. Figure shows the absolute change in KI67 between the diagnostic core biopsy and the excision/pre-operative repeat core biopsy

There were 47 patients in whom Ki67 was assessed following BrET, either via repeat core biopsy prior to surgery or on the surgical excision (Fig. 4; Table 5). When patients were categorised based on their change in Ki67 as per the POETIC trial [16], most patients (*n* = 26, 55.3%) were in the High–Low category, where they started with a high Ki67 (≥ 10%), and dropped into the Low Ki67 group (< 10%). The remaining patients were the Low–Low (*n* = 14), Low–High (*n* = 1), and High–High (*n* = 6) categories accordingly. When examining the absolute changes in Ki67, there was an overall decrease in mean Ki67 from 19 to 7% with BrET. There was no correlation between this absolute reduction in Ki67, and time on BrET in days.

**Table 5:** Change in Ki67 against time on BrET

Time on BrET	Ki67 score^a^				
	L–L	L–H	H–H^b^	H–L^b^	Total
Up to 1 month	2	0	1 (16.6%)	5 (83.3%)	8
Up to 2 months	4	0	1 (35.0%)	3 (75.0%)	8
Up to 3 months	5	0	2 (11.8%)	15 (88.2%)	22
More than 3 months	3	1	2 (40%)	3 (60%)	9
Total	14	1	6	26	47

^a^Ki67 scores were dichotomised and patients divided into four groups as follows: low–low (Ki67 before and after < 10%); high–low (Ki67 before ≥ 10%, Ki67 after < 10%); high–high (Ki67 before and after ≥ 10%); and low–high (Ki67 before < 10%, Ki67 after ≥ 10%)

^b^Percentages in parenthesis summarise the change in Ki67 in patients that had a high baseline (Ki67 ≥ 10%)

## Discussion

The B-MaP-C study demonstrated that the management of patients presenting with primary breast cancer in the UK during the peak (Alert level 4) phase of the pandemic was, overall, in keeping with pre-covid standards. Recognising the prospect of probable limited theatre availability, and the need to deliver a priority-driven level of care, guidelines were published by the Association of Breast Surgery (ABS) and other national and international bodies [1, 4–6, 9–11]. In the absence of neo-adjuvant chemotherapy, prioritisation for surgical treatment was given to premenopausal women with triple negative or HER2 positive breast cancers. Post-menopausal women with ER-positive breast cancer were advised to commence bridging endocrine treatment, until such a time would arise that theatre capacity had improved and the COVID-19 associated surgical risks had been established. Although BrET was used, outside of routine pre-covid practice, there was a rapid reduction in patients receiving BrET, within 1 month (mid-April) of the start of the pandemic to nearer pre-pandemic practice being resumed by 4 months (Fig. 1). This transition in early April 2020 towards standard management decisions occurred before the number of positive COVID cases began to resolve (early May 2020). This was achieved due to a combination of increasing theatre capacity in a relatively ‘COVID safe’ setting, referral triaging and a reduction in volume of symptomatic new patient clinics alongside pausing of the breast screening programme.

The advice to start BrET for patients with ER-positive breast cancers, was aimed to relieve pressure on theatre capacity, to allow prioritisation of patient groups who could not have their surgical treatment delayed safely, and to postpone surgery to a time when risks of infection were better managed with appropriate theatre pathways. Reassuringly, the majority of patients placed on BrET (95.6%; Table 1) were strongly ER positive suggesting this strategy was reserved for those with tumours that were most likely to respond. However, some pre-/perimenopausal women were started on aromatase inhibitor therapy without LHRH analogue It is possible that the use of AI (± Goserelin) in this age group was due to thromboembolic considerations with Tamoxifen, particularly with the potential need to schedule surgery at short notice, but we suspect more likely reflects inaccurate menopausal status documentation and therefore uncertainty in data collection but not clinical management regarding menopausal status, leading to the categorisation as ‘peri-menopause’.

Prior to the pandemic, there was infrequent utilisation of neoadjuvant ET (NET); with only 1.5% of patients diagnosed with breast cancer thing the UK breast screening programme receiving NET [20], and there is considerable variation in practice nationally and internationally. The UK NeST study demonstrated that only 14% of systemic therapy given in the neoadjuvant setting was ET despite its relative safety and tolerability compared to chemotherapy [21, 22]. The general reluctance to use NET may be driven by lack of evidence, particularly in pre-menopausal women where the evidence and subsequent guidance is less clear. The evidence around the safety and efficacy of neoadjuvant endocrine treatment compared to primary surgery is limited including regarding optimal treatment duration, although a small RCT suggested minimal differences between 4 and 6 months [23].

In this study, a change in surgical plan from planned mastectomy at the start of BrET, to BCS occurred in 35.2% of patients (97/275) after a median of 64 (IQR [37, 107]) days of BrET. Comparatively, a small, multicenter, prospective, longitudinal study [24] reported on the optimal duration of neoadjuvant letrozole that would downstage disease to accommodate BCS (139 patients). Of the 69% of patients that became eligible for BCS, the median time on NET was 7.5 months (95% CI 6.3–8.5 months). Letrozole was well-tolerated, with mostly mild-to-moderate (grade 1–2) adverse events. Similarly, a previous study of 182 patients concluded that the use of NET beyond 3 months increased the conversion to BCS from an initial requirement for mastectomy from 60% at 3 months to 72% beyond this [25]. The ACOSOG Z1031 study [26] reported downstaging to accommodate BCS in 51% of 374 patients after 16 to 18 weeks of NET. The majority of cases not undergoing BCS despite downstaging were due to patient choice (12/17). The TEAM IIA trial [27] examined the clinical and radiological response to 6 months of neoadjuvant exemestane in 102 post-menopausal, strongly ER-positive patients. A clinically measurable response was seen in 58.7% patients at 3 months and 68.3% at > 3 months, with an improved feasibility of BCS from 61.8 to 70.6% (*p* = 0.012). In a subsequent meta-analysis of 1452 patients from seven RCTs, the feasibility of BCS increased from 43.3 to 60.4% (*p* < 0·001), but BCS was performed in only 51.8%. The reluctance not to proceed with BCS despite feasibility to do so was driven by the pre-NET clinical assessment, tumour multicentricity and tumour size [28].

As the median length of BrET was relatively short in this study, there was little reduction in size on final pathology compared to baseline imaging in the overall cohort, which was only appreciable following 3 months of treatment. This difference was a median of 4 mm [IQR − 20, 4], based on the assumption of accurate sizing by USS. The results reported in this study regarding the stated changes in surgical plans should be interpreted with some caution, since, we could not exclude that considerations of unavailability or perceived unavailability of radiotherapy and/or re-excision capacity might have impacted on the baseline stated plan for mastectomy. In addition, due to ‘pooled lists’, there may have an impact onto surgical decision-making. The modest tumour size reductions, and mean baseline tumour size of 21 mm for those deemed to require mastectomy also suggest caution in interpretation and we have therefore not explicitly used the term ‘surgical downstaging’ but have reported the stated changes in treatment plan from baseline to that post-BrET prior to surgery.

Only a small proportion of patients in this study, had serial Ki67 measurement, limiting any broader conclusions from these results. The recent publication on long-term outcome and prognostic value of Ki67 after perioperative endocrine therapy in post-menopausal women reported on a higher risk of disease recurrence in patients with persistent high Ki67 score suggesting a possible benefit of additional adjuvant therapies [16]. The ability to downstage disease, with a tolerable and safe approach, along with the ability to incorporate a proven prognostic biomarker, makes pre-operative endocrine therapy an attractive option. The length of treatment remains unknown, and whether this should be 14 days of neo-adjuvant endocrine therapy to identify patients of higher risk, who would require additional therapy, or potentially 3 months for the added benefit of downstaging disease and accommodating breast conservation. To investigate the former, the POETIC-A trial [29] will aim to randomise patients with persistently high Ki67 following a short treatment window, to additional CDK4/6 inhibitor therapy.

This observational study has limitations. The length of time on BrET could be prone to reporting inaccuracy or recall bias. The date started might be the date the patient received the prescription from the clinician, and not when the patient actually commenced treatment, and drug compliance was not assessed. This variability differs to randomised controlled trials where the start of treatment is stipulated, reporting is standardised, and the exact treatment duration is documented. With regards to Tamoxifen, the practice of stopping it two weeks prior to surgery was not accounted for in this study, due to the variability in practice. There was a high number of patients (35%) in this study with a planned mastectomy who had a change in surgical plan to BCS following BrET. This is subject to bias, as the surgical decision-making prior to initiation of BrET would have been driven by the pressures of the pandemic, and may be due to apprehension around radiotherapy availability and risk of margin re-excision during the peak of the pandemic. Future work should examine trends in use of BrET post-pandemic.

Within this study, BrET was found to be tolerable, and safe, overall, as evidenced by the fact that only 1.2% of patients (12/990) required expedited surgery due to lack of response, and only 0.8% (8/990) where surgery had to be expedited due to lack of tolerance/compliance with ET. There were 8.2% (81/990) in whom ET was continued as the primary treatment approach, mostly due to patients declining surgery (31/79, Table 2). Overall drops in cellular proliferation (Ki67) occurred relatively quickly and there were definite but small reductions in median tumour size, apparent even after short-term treatment and increasing with treatment duration. The results of this study, as well as those summarised above, would support a longer period (at least 3 months) of BrET to impact surgical outcomes, however in this relatively limited study, it is not possible to infer the long-term oncological implications. EndoNET is a randomised controlled trial currently in the set-up phase which will evaluate if neo-adjuvant endocrine treatment increases breast conservation rates and/or quality of life. Post-menopausal patients with early ER+ breast cancer will be randomised to standard primary surgery versus 6 months of neo-adjuvant endocrine treatment followed by surgery [30].

## Conclusions

In conclusion, short course BrET is safe, effective, well-tolerated, and has the potential to provide valuable prognostic information from serial Ki67 assessment. There was no associated adverse impact on surgical decision-making. This evidence, taken together with clinical trial data [16] supports the routine use of short-term pre-op ET to allow assessment of endocrine sensitivity, and justifies the use of this approach in situations where theatre access is constrained, as occurred during the pandemic.

### Electronic supplementary material

Below is the link to the electronic supplementary material.

Supplementary file1 (DOCX 44 kb)

## Data Availability

Anonymised patient data is the property of the collaborating unit, and has been entered onto a secure server for the purpose of this study. This data is not accessible to those who do have ownership, and is not availble to request.

## Abbreviations

BrET: Bridging endocrine therapy
QoL: Quality of life
RT: Radiotherapy
ET: Endocrine therapy
5F RT: 5 Fraction radiotherapy
ER: Oestrogen-receptor
DCIS: Ductal carcinoma in situ
IQR: Interquartile range
NET: Neoadjuvant endocrine therapy
