# Supplementary material for: Bridging pre-surgical endocrine therapy for breast cancer during the COVID-19 pandemic: outcomes from the B-MaP-C study
Source: Breast Cancer Res Treat. 2023 Apr 3;199(2):265–79. doi: 10.1007/s10549-023-06893-4 (PMC10068712; doi:10.1007/s10549-023-06893-4)
Supplement: Supplementary file 1 — Supplementary file1 (DOCX 44 kb) [file 10549_2023_6893_MOESM1_ESM.docx]

|  | Anastrozole (of which N had Goserelin) | Letrozole (of which N had Goserelin) | Exemestane  (of which N had Goserelin) | Tamoxifen  (of which N had Goserelin) | Goserelin alone |
| --- | --- | --- | --- | --- | --- |
| Pre & peri menopausal  (n=179) | 2 (0) | 35 (12) | 1 (0) | 132 (6) | 9 |
| Post-menopausal  (n=867) | 87 (1) | 736 (+ 2) | 6 ( 0) | 37 (0) | 1 |
| Unknown | 6 | 27 | 0 | 9 | 0 |

**Table S1. Prescribed bridging endocrine therapy, based on menopausal status**
